# Serum levels of matrix metalloproteinase 2 and matrix metalloproteinase 9 elevated in polypoidal choroidal vasculopathy but not in age-related macular degeneration

**Published:** 2013-03-21

**Authors:** Renpan Zeng, Feng Wen, Xiongze Zhang, Yu Su

**Affiliations:** State Key Laboratory of Ophthalmology, Zhongshan Ophthalmic Center, Sun Yat-sen University, Guangzhou, China

## Abstract

**Purpose:**

Age-related macular degeneration (AMD) and polypoidal choroidal vasculopathy (PCV) are the leading causes of vision loss in the elderly Asian population. Previous studies have confirmed that abnormal extracellular matrix (ECM) metabolism plays an important role in the pathogenesis of AMD and PCV. However, the dynamic metabolism of the ECM is closely regulated by matrix metalloproteinases (MMPs) and tissue metalloproteinase inhibitors (TIMPs). Whether MMPs and TIMPs participate in the pathogenesis of AMD and PCV remains unclear. The aim of this study was to investigate the correlation between circulating MMP and TIMP levels and AMD and PCV.

**Methods:**

The serum levels of MMPs (MMP1, MMP2, MMP3, and MMP9) and TIMPs (TIMP1 and TIMP3) were quantified using enzyme-linked immunosorbent assays in four groups of subjects (n=342): early AMD (group 1, n=75), neovascular AMD (group 2, n=89), PCV (group 3, n=98), and age- and gender-matched controls (group 4, n=80).

**Results:**

The mean concentrations of the two gelatinases, MMP2 and MMP9, in the PCV group were significantly higher than that of the control (p=0.001, p<0.001, respectively), early AMD (both p<0.001), and neovascular AMD (p=0.005, p=0.001, respectively) groups. Moreover, the serum MMP2 concentration was positively correlated with the serum MMP9 concentration in the PCV group (r=0.822, p<0.001). However, the mean concentrations of MMP2 and MMP9 in the early AMD and neovascular AMD groups were not significantly different from that of the control group (p>0.05). The mean serum levels of MMP1, MMP3, TIMP1, and TIMP3 were not significantly different among the four groups.

**Conclusions:**

This pilot study first reveals a link between increased levels of circulating gelatinases (MMP2 and MMP9) and PCV but not AMD, which may provide a biologically relevant marker of ECM metabolism in patients with PCV. This finding suggests that the two disorders may have different molecular mechanisms.

## Introduction

Age-related macular degeneration (AMD) and polypoidal choroidal vasculopathy (PCV) are the leading causes of blindness in the elderly Asian population [1-3]. Early AMD is characterized by drusen and retinal pigmentary changes that predict the risk for advanced AMD [4]. Neovascular AMD (nAMD) is the main type of advanced AMD and is characterized by typical choroidal neovascularization (CNV) [5]. PCV has been recognized as an abnormal choroidal vasculopathy distinct from typical CNV [6,7]. Both nAMD and PCV can cause severe and rapid vision loss due to recurrent retinal exudation, subretinal hemorrhage, and serosanguineous detachment of the retinal pigment epithelium (RPE) [8,9]. The etiology and pathogenesis of AMD and PCV have not been fully elucidated.

Previous studies have confirmed that abnormal extracellular matrix (ECM) metabolism plays an important role in the pathogenesis of AMD and PCV [10-12]. Bruch’s membrane (BM) is an elastin- and collagen-rich ECM strategically located between the RPE and the fenestrated choroidal capillaries of the eye. Histopathological studies have shown that the ECM components (e.g., collagen layer and elastic layer) of BM change its thickness and integrity in eyes with AMD; diffuse and focal thickening of BM is considered a sign of early AMD [13], while disruption and segmental thinning of BM can be observed at the site of CNV in nAMD [14-16]. In addition, drusen are abnormal deposits of ECM located between the RPE and BM, the main sign of early AMD, and soft and large drusen are risk factors for progression to advanced AMD [17]. For PCV, a recent study [18] demonstrated that increased expression of the human serine protease HTRA1, which possesses elastase activity, in the mouse RPE induces the cardinal features of PCV (polypoidal vascular dilations and a network of branching abnormal choroid vessels). An ultrastructural analysis of the mouse showed marked attenuation of the choroidal vessels and severe degeneration of the elastic laminae and the tunica media of choroidal vessels [18]. These features were similar to the histopathologic findings from surgically excised human PCV specimens [12]. The authors speculated that other enzymes related to ECM metabolism in the choroid are also involved in the pathogenesis of PCV.

Abnormal ECM metabolism is involved in AMD and PCV. Alterations of the ECM components lead to structural and functional changes in BM and the choroidal vessel wall. However, the dynamic metabolism of the ECM is closely regulated by matrix metalloproteinases (MMPs) and tissue metalloproteinase inhibitors (TIMPs) [19]. The circulating MMPs and TIMPs have been suggested to control aspects of vascular remodeling and angiogenesis [20]. We hypothesize that the circulating MMP and TIMP imbalance affecting ECM metabolism may contribute to the pathogenesis of AMD and PCV. However, the effects of MMPs and TIMPs on AMD and PCV have not been well investigated. The aim of this study was to investigate the correlation between the levels of circulating MMPs and TIMPs and AMD and PCV.

## Methods

### Study participants

All study participants were Han Chinese individuals recruited from March 2012 to December 2012 at the Zhongshan Ophthalmic Center of Sun Yat-sen University. The study protocol was approved by the institutional review board at the Zhongshan Ophthalmic Center of Sun Yat-sen University and followed the tenets of the Declaration of Helsinki. Informed consent was obtained from all study participants, who were fully informed about the purpose and procedures of this study.

All subjects (age ≥50 years) underwent a complete ophthalmic examination that included visual acuity measurements, slit-lamp biomicroscopy, and a retinal examination including ophthalmoscopy and color fundus photography after pupil dilation. Fluorescein angiography (FFA) and indocyanine green angiography (ICGA) were performed if there was a clinical suspicion of nAMD or PCV. The fundus photographs and FFA and ICGA images of these subjects were randomized and reviewed by at least two of the authors. The authors were blinded to the age and clinical history of the participants. Early AMD was diagnosed similarly to the international AMD classification and grading system by the presence of either soft indistinct drusen (≥63 μm) only or any drusen combined with pigmentary abnormalities in the macular area [21] with no geographic atrophy or exudative lesions [22]. nAMD was diagnosed by the identification of typical CNV using FFA or ICGA. PCV was diagnosed in the presence of characteristic polypoidal lesions with a continuous branching choroidal vascular network using ICGA [2,3]. If the diagnosis differed between the two eyes, the subject was categorized according to the severity of the changes in the worse eye. Cases diagnosed as probable, patients with nAMD and PCV in the same eye [23], and patients who had nAMD in one eye and PCV in the other were excluded. Patients with other neovascularized maculopathies, such as retinal angiomatous proliferation, angioid streaks, pathological myopia, presumed ocular histoplasmosis, and other retinal or choroidal diseases that could account for CNV were also excluded. The medical records of all patients were reviewed. Eyes that had received any treatment (such as laser photocoagulation, photodynamic therapy, intravitreal injections of steroids, or anti-vascular endothelial growth factor) or surgery before the start of this study were excluded.

All control subjects were unrelated to the case subjects and were aged ≥50 years. All patients underwent comprehensive ophthalmic examinations, and those with macular changes (such as drusen or pigment abnormalities), macular degeneration due to any cause, or refracting media opacities preventing clear visualization of the fundus in either eye were excluded from recruitment. The study also excluded patients for the following: a history of systemic diseases (including hypertension, diabetes) or infectious or autoimmune diseases; a history of neoplastic, hepatic, lung, or cardiovascular diseases, including coronary artery disease, stroke, and peripheral arterial disease; or any surgical procedure in the preceding 6 months.

### Serum collection

Venous samples were collected into serum separator tubes, clotted (approximately 30 min) at room temperature and then centrifuged at 1000 ×g for 15 min. The serum samples were transferred to a fresh tube and kept frozen at −80 °C. When used for the MMP and TIMP measurements, the serum samples were thawed on ice. The serum samples were randomized so that the investigator who analyzed the samples was blinded to the clinical history of the subjects.

### Measurement of serum MMP and TIMP concentrations

Serum levels of MMPs (MMP1, MMP2, MMP3, MMP9) and TIMPs (TIMP1 and TIMP3) were quantified with a sandwich enzyme-linked immunosorbent assay (ELISA) using the (human) ELISA kit (Abnova Ltd, Taipei, Taiwan) for each MMP and TIMP. These assays were processed according to the manufacturer’s instructions. All samples were diluted according to the instructions and measured in duplicate. The results were averaged as the mean±standard deviation (SD).

### Statistical methods

Statistical analysis of the data was performed using the statistical package for social sciences (SPSS) software package (version 16.0, SPSS Inc., Chicago, IL). Baseline characteristics were compared among the four groups using a one-way analysis of variance (ANOVA) with a post-hoc Scheffe’s test for mean age and a χ^2^ test for the gender proportion. The differences in MMPs and TIMPs among the four groups were evaluated using a one-way ANOVA with a post-hoc Scheffe’s test. Pearson’s correlation test was used to analyze the correlation between serum MMP2 and MMP9 concentrations in the PCV group. For all tests, a p value of less than 0.05 was considered statistically significant.

## Results

### Characteristics of the subjects

Serum samples were recruited from 342 subjects, including 75 with early AMD, 89 with nAMD, 98 with PCV, and 80 controls. There were no significant differences in average age or gender distribution among the four groups (Table 1).

**Table 1:** Characteristics of subjects in the four groups

Characteristics	early AMD (n=75)	nAMD (n=89)	PCV (n=98)	Control (n=80)
Gender (female/male) *	36/39	39/50	40/58	36/44
Mean age (years) ^†^	63.9±11.9	64.2±11.0	62.1±10.2	62.0±9.5
Age range (years)	50–93	50–85	50–82	50–81

Abbreviations: AMD=Age-related macular degeneration; nAMD=neovascular Age-related macular degeneration; PCV=Polypoidal choroidal vasculopathy * χ^2^ test. The differences in the gender distribution in each of the 4 groups were not statistically significant (χ^2^=0.38, p=0.944). ^†^ One-Way ANOVA test. The differences in the mean age in each of the 4 groups were not statistically significant (F=0.773, p=0.511).

### Serum levels of MMPs and TIMPs

**Figure 1:**
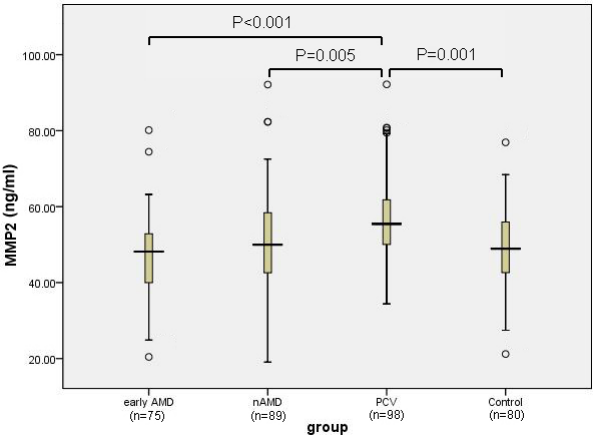
MMP2 levels in the serum from early AMD (n=75), nAMD (n=89), PCV (n=98), and control subjects (n=80) were measured with ELISA. One-way ANOVA with a post-hoc Scheffe test demonstrated that the serum concentration of MMP2 in the PCV group was significantly higher than that of the control (p=0.001), nAMD (p=0.005), and early AMD groups (p<0.001). Abbreviations: AMD=Age-related macular degeneration; nAMD=neovascular Age-related macular degeneration; PCV=Polypoidal choroidal vasculopathy; ELISA=Enzyme-linked immunosorbent assay.

**Figure 2:**
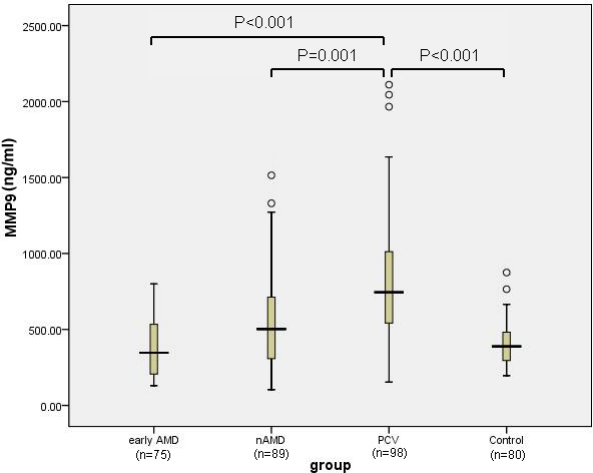
MMP9 levels in the serum from early AMD (n=75), nAMD (n=89), PCV (n=98), and control subjects (n=80) were measured with ELISA. One-way ANOVA with a post-hoc Scheffe test demonstrated that the serum concentration of MMP9 in the PCV group was significantly higher than that of the control (p<0.001), early AMD (p<0.001), and nAMD groups (p=0.001). Abbreviations: AMD=Age-related macular degeneration; nAMD=neovascular Age-related macular degeneration; PCV=Polypoidal choroidal vasculopathy; ELISA=Enzyme-linked immunosorbent assay.

**Figure 3:**
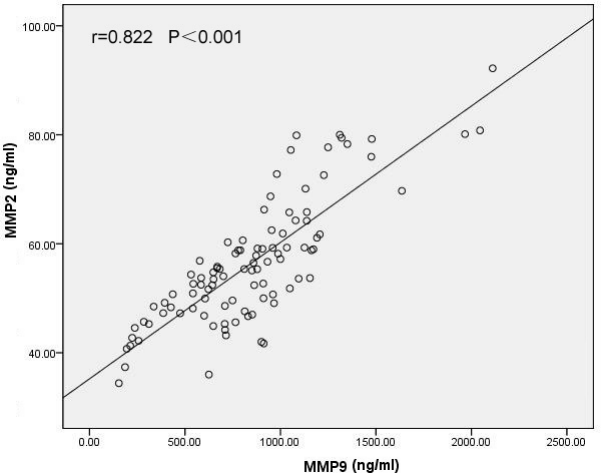
Correlation analysis showing that the serum MMP2 concentrations were positively correlated with serum MMP9 concentrations in the PCV group (r=0.822, p<0.001). Pearson’s correlation test was used (p<0.05 significance; r=correlation coefficient).

The serum levels of the MMPs and TIMPs in the four groups are shown in Table 2. The differences in the mean concentration of MMP1 (p=0.33), MMP3 (p=0.417), TIMP1 (p=0.594), and TIMP3 (p=0.311) in each group were not statistically significant. In contrast, the mean MMP2 concentration in the PCV group was significantly higher than that of the control (p=0.001), early AMD (p<0.001), and nAMD (p=0.005) groups, as shown in Figure 1. The mean MMP9 concentration in the PCV group was significantly higher than that of the control (p<0.001), early AMD (p<0.001), and nAMD (p=0.001) groups, as demonstrated in Figure 2. Moreover, the serum MMP2 concentration was positively correlated with the serum MMP9 concentration in the PCV group (r=0.822, p<0.001), as shown in Figure 3. However, the mean concentrations of MMP2 and MMP9 in the early AMD and nAMD groups were not significantly different from those of the control group (p>0.05).

**Table 2:** The serum levels of MMPs and TIMPs in the four groups (ng/ml)

Serum factors (Mean ± SD)	early AMD (n=75)	nAMD (n=89)	PCV (n=98)	Control (n=80)
MMP1	631.7±352.4*	643.7±441.9*	694.6±518.9*	658.3±389.5
MMP2	47.6±12.6*	50.9±14.7*	56.9±11.1^†^	49.0±10.8
MMP3	15.1±7.9*	16.1±17.2*	18.2±17.3*	17.9±18.8
MMP9	388.0±199.4*	579.6±353.9*	801.4±424.7^‡^	420.7±162.0
TIMP1	190.2±189.5*	209.7±247.8*	215.9±218.9*	195.8±218.6
TIMP3	39.3±18.7*	42.0±16.2*	40.2±25.4*	36.7±19.5

Abbreviations: MMP=Matrix metalloproteinases; TIMP=Tissue metalloproteinase inhibitors; AMD=Age-related macular degeneration; nAMD=neovascular Age-related macular degeneration; PCV=Polypoidal choroidal vasculopathy; SD=standard deviation compared with control: * p>0.05; ^†^ p<0.05; ^‡^ p<0.001

## Discussion

Recent studies have focused on the biologic abnormalities of the ECM in AMD [10] and PCV [12]. The ECM is a dynamic structure and primarily regulated by the balance between MMPs and TIMPs [24]. MMPs are zinc-dependent endopeptidases belonging to a family of proteases known as metzincins, and degrade most ECM constituents [19], while TIMPs are major endogenous regulators of MMPs in tissue. TIMPs bind with MMPs in a 1:1 ratio to form binary noncovalent complexes that block substrate cleavage binding sites [25].

In the present pilot study, we investigated the systemic levels of MMPs (MMP1, MMP2, MMP3, MMP9) and TIMPs (TIMP1 and TIMP3) in patients with early AMD, nAMD, and PCV and normal control subjects. All these factors can be secreted by RPE cells and choroidal vascular endothelial cells and are expressed in BM [26-28]. We found that MMP2 and MMP9 were significantly elevated in the PCV group and positively correlated with each other. MMP2 and MMP9 are the only two gelatinases in humans. Disequilibrium of the two gelatinases suggests a role of abnormal ECM metabolism in PCV. Meanwhile, the results indicate that the circulating MMP2 and MMP9 act synergistically in the occurrence and development of PCV, and the elevated levels of circulating MMPs may provide a biologically relevant marker of ECM metabolism for patients with PCV. Furthermore, our findings prove the speculation that except HTRA1, other enzymes such as MMPs and TIMPs, related to ECM metabolism in the choroid may be involved in the pathogenesis of PCV [18]. Finally, the elevated serum levels of the MMPs indicate that PCV may be a type of systemic vascular disease, such as atherosclerosis or vascular aneurysm. In fact, ICGA demonstrates that the polypoidal lesions of PCV have an aneurismal shape. Histopathologic findings demonstrate notable arteriosclerotic changes in PCV lesions and disruption of the elastic layer within the wall of polypoidal vessels [12,29], which is known to decrease the elasticity and strength of the vascular wall, leading to aneurysm formation. A previous study reported that fluctuations in the equilibrium of circulating MMPs and TIMPs contribute to diseases including atherosclerosis and vascular aneurysm [30].

We speculated that the association between serum MMP2 and MMP9 and PCV may largely attribute to their proteolytic effects on the ECM proteins (elastin, collagen), allowing the breakdown of the basement membrane of choroidal vessels and BM. Such changes may ultimately contribute to weaken the vascular wall and cause vessel dilatation (forming polypoidal lesions) and break through the BM into the sub-RPE space. Particular interest has been focused on MMP9 due to its ability to degrade basement membrane components such as type IV collagen [31]. In addition, MMP2 and MMP9 have additional inhibitory effects on the Ca^2+^-dependent mechanism of vascular smooth muscle (VSM) contraction [32], which induces blood vessel relaxation and progressive dilatation [20]. VSM contraction may have a role in the early formation of aneurismal polypoidal lesions. Thus, the MMP-induced inhibition of VSM contraction may function synergistically with MMP degradation of the ECM and thus contribute to further weakening of the blood vessel wall and polypoidal lesion formation.

However, we did not found any associations between the serum levels of the MMPs and TIMPs and early AMD and nAMD. Previous studies have indicated that MMPs and TIMPs play important roles in early AMD [33] and nAMD [34-36], but are more likely to be imbalanced at a local tissue in the eye, similar to vascular endothelial growth factor (VEGF), which is elevated in the aqueous humor and vitreous of patients with nAMD [37] but not in the serum [38]. Interestingly, a recent study [36] reported that MMP9 was significantly elevated in the aqueous humor of patients with nAMD (p=0.004) and that macular thickness was significantly associated with the MMP9 concentration (p<0.001).

The different findings in PCV and AMD also suggest the two disorders may have different molecular mechanisms. Although PCV and nAMD share some similar clinical manifestations, the diseases’ treatment effects, visual prognosis, and genetic background are somewhat different [39,40]. We hypothesize that PCV may be a systemic disease concerned with systemic imbalance of MMPs and TIMPs, whereas AMD is a localized disease with localized imbalance of MMPs and TIMPs in the eye. In addition, abnormal ECM metabolism caused by MMP and TIMP disequilibrium might play a more important role in PCV than in nAMD. The *HTRA1* gene polymorphisms are associated with the susceptibility of AMD and PCV [41]. However, overexpression of HTRA1 protein, human serine protease, in mice RPE caused PCV but not AMD, and the degeneration of the choroid vessels was much more severe than that of BM [18]. Moreover, our previous genetic study showed that a common variant (rs10757278) on chromosome 9p21 was associated with PCV but not with nAMD in a Chinese population [42]. Chromosome 9p21 plays an important role in vascular ECM remodeling; variants on 9p21 have been shown to be closely related to the susceptibility to intracranial aneurysm, abdominal aortic aneurysm, and coronary artery disease [43]. However, we recently found that rs42524 in the collagen type 1 alpha-2 (*COL1A2*) gene is associated with nAMD but not with PCV [44]. Taken together, these findings may indicate that genes related to ECM affect susceptibility to PCV and nAMD differently.

The main limitations of this pilot study include the relatively small sample size and the exclusion of some MMPs and TIMPs from the analysis. These results need to be confirmed in a large cohort and with a more comprehensive investigation of all MMPs and TIMPs. In addition, total MMP (proenzyme and active forms) levels were measured in this study. All MMPs require activation by zymogens to become active enzymes. The antibodies used in the ELISA kits cannot distinguish the active forms from their proenzyme forms. The zymogens lack activity, and TIMPs may block activated MMPs. Therefore, the increased MMP2 and MMP9 immunoreactivity observed in this study does not necessarily correspond to augmented enzymatic activity, and advanced techniques for measuring MMP activity, particularly in vivo, are needed.

In summary, in this pilot study, we first reveal an association between elevated MMP2 and MMP9 levels in the circulation and PCV, but not AMD. It is difficult to draw wide-ranging conclusions or assumptions based on these observations. However, this is an important starting point. Future, large-scale studies are required to clarify these findings, including the link with systemic ECM metabolism.

## References

[1] Li Y, Xu L, Wang YX, You QS, Yang H, Jonas JB (2008). Prevalence of age-related maculopathy in the adult population in China: The Beijing Eye Study.. Am J Ophthalmol.

[2] Wen F, Chen C, Wu D, Li H (2004). Polypoidal choroidal vasculopathy in elderly Chinese patients.. Graefes Arch Clin Exp Ophthalmol.

[3] Liu Y, Wen F, Huang S, Luo G, Yan H, Sun Z, Wu D (2007). Subtype lesions of neovascular age-related macular degeneration in Chinese patients.. Graefes Arch Clin Exp Ophthalmol.

[4] Lim LS, Mitchell P, Seddon JM, Holz FG, Wong TY (2012). Age-related macular degeneration.. Lancet.

[5] Zeng R, Wen F, Zhang X, Zuo C, Li M, Chen H, Wu K (2012). An rs9621532 variant near the TIMP3 gene is not associated with neovascular age-related macular degeneration and polypoidal choroidal vasculopathy in a Chinese Han population.. Ophthalmic Genet.

[6] Yannuzzi LA, Ciardella A, Spaide RF, Rabb M, Freund KB, Orlock DA (1997). The expanding clinical spectrum of idiopathic polypoidal choroidal vasculopathy.. Arch Ophthalmol.

[7] Li M, Wen F, Zuo C, Zhang X, Chen H, Huang S, Luo G (2010). SERPING1 polymorphisms in polypoidal choroidal vasculopathy.. Mol Vis.

[8] de Jong PT (2006). Age-related macular degeneration.. N Engl J Med.

[9] Wu K, Wen F, Zuo C, Li M, Zhang X, Chen H, Zeng R (2012). Lack of association with PEDF Met72Thr variant in neovascular age-related macular degeneration and polypoidal choroidal vasculopathy in a Han Chinese population.. Curr Eye Res.

[10] Chong NH, Keonin J, Luthert PJ, Frennesson CI, Weingeist DM, Wolf RL, Mullins RF, Hageman GS (2005). Decreased thickness and integrity of the macular elastic layer of Bruch's membrane correspond to the distribution of lesions associated with age-related macular degeneration.. Am J Pathol.

[11] Spraul CW, Lang GE, Grossniklaus HE, Lang GK (1999). Histologic and morphometric analysis of the choroid, Bruch's membrane, and retinal pigment epithelium in postmortem eyes with age-related macular degeneration and histologic examination of surgically excised choroidal neovascular membranes.. Surv Ophthalmol.

[12] Nakashizuka H, Mitsumata M, Okisaka S, Shimada H, Kawamura A, Mori R, Yuzawa M (2008). Clinicopathologic findings in polypoidal choroidal vasculopathy.. Invest Ophthalmol Vis Sci.

[13] Ramrattan RS, van der Schaft TL, Mooy CM, de Bruijn WC, Mulder PG, de Jong PT (1994). Morphometric analysis of Bruch's membrane, the choriocapillaris, and the choroid in aging.. Invest Ophthalmol Vis Sci.

[14] Grossniklaus HE, Miskala PH, Green WR, Bressler SB, Hawkins BS, Toth C, Wilson DJ, Bressler NM (2005). Histopathologic and ultrastructural features of surgically excised subfoveal choroidal neovascular lesions: submacular surgery trials report no. 7.. Arch Ophthalmol.

[15] Spraul CW, Lang GE, Grossniklaus HE, Lang GK (1998). Characteristics of drusen and changes in Bruch's membrane in eyes with age-related macular degeneration. Histological study.. Ophthalmologe.

[16] van der Schaft TL, Mooy CM, de Bruijn WC, Oron FG, Mulder PG, de Jong PT (1992). Histologic features of the early stages of age-related macular degeneration. A statistical analysis.. Ophthalmology.

[17] Wang JJ, Foran S, Smith W, Mitchell P (2003). Risk of age-related macular degeneration in eyes with macular drusen or hyperpigmentation: the Blue Mountains Eye Study cohort.. Arch Ophthalmol.

[18] Jones A, Kumar S, Zhang N, Tong Z, Yang JH, Watt C, Anderson J (2011). Amrita, Fillerup H, McCloskey M, Luo L, Yang Z, Ambati B, Marc R, Oka C, Zhang K, Fu Y. Increased expression of multifunctional serine protease, HTRA1, in retinal pigment epithelium induces polypoidal choroidal vasculopathy in mice.. Proc Natl Acad Sci USA.

[19] Lim CS, Shalhoub J, Gohel MS, Shepherd AC, Davies AH (2010). Matrix metalloproteinases in vascular disease–a potential therapeutic target?. Curr Vasc Pharmacol.

[20] Raffetto JD, Khalil RA (2008). Matrix metalloproteinases and their inhibitors in vascular remodeling and vascular disease.. Biochem Pharmacol.

[21] Chau KY, Sivaprasad S, Patel N, Donaldson TA, Luthert PJ, Chong NV (2008). Plasma levels of matrix metalloproteinase-2 and −9 (MMP-2 and MMP-9) in age-related macular degeneration.. Eye (Lond).

[22] Bird AC, Bressler NM, Bressler SB, Chisholm IH, Coscas G, Davis MD, de Jong PT, Klaver CC, Klein BE, Klein R, Mitchell P, Sarks JP, Sarks SH, Soubrane G, Taylor HR, Vingerling JR (1995). An international classification and grading system for age-related maculopathy and age-related macular degeneration. The International ARM Epidemiological Study Group.. Surv Ophthalmol.

[23] Chen Y, Wen F, Sun Z, Wu D (2008). Polypoidal choroidal vasculopathy coexisting with exudative age-related macular degeneration.. Int Ophthalmol.

[24] Malemud CJ (2006). Matrix metalloproteinases (MMPs) in health and disease: an overview.. Front Biosci.

[25] Zitka O, Kukacka J, Krizkova S, Huska D, Adam V, Masarik M, Prusa R, Kizek R (2010). Matrix metalloproteinases.. Curr Med Chem.

[26] Guo L, Hussain AA, Limb GA, Marshall J (1999). Age-dependent variation in metalloproteinase activity of isolated human Bruch's membrane and choroid.. Invest Ophthalmol Vis Sci.

[27] Eichler W, Friedrichs U, Thies A, Tratz C, Wiedemann P (2002). Modulation of matrix metalloproteinase and TIMP-1 expression by cytokines in human RPE cells.. Invest Ophthalmol Vis Sci.

[28] Della NG, Campochiaro PA, Zack DJ (1996). Localization of TIMP-3 mRNA expression to the retinal pigment epithelium.. Invest Ophthalmol Vis Sci.

[29] Kuroiwa S, Tateiwa H, Hisatomi T, Ishibashi T, Yoshimura N (2004). Pathological features of surgically excised polypoidal choroidal vasculopathy membranes.. Clin Experiment Ophthalmol.

[30] Hobeika MJ, Thompson RW, Muhs BE, Brooks PC, Gagne PJ (2007). Matrix metalloproteinases in peripheral vascular disease.. J Vasc Surg.

[31] Hadler-Olsen E, Fadnes B, Sylte I, Uhlin-Hansen L, Winberg JO (2011). Regulation of matrix metalloproteinase activity in health and disease.. FEBS J.

[32] Chew DK, Conte MS, Khalil RA (2004). Matrix metalloproteinase-specific inhibition of Ca2+ entry mechanisms of vascular contraction.. J Vasc Surg.

[33] Leu ST, Batni S, Radeke MJ, Johnson LV, Anderson DH, Clegg DO (2002). Drusen are Cold Spots for Proteolysis: Expression of Matrix Metalloproteinases and Their Tissue Inhibitor Proteins in Age-related Macular Degeneration.. Exp Eye Res.

[34] Ebrahem Q, Qi JH, Sugimoto M, Ali M, Sears JE, Cutler A, Khokha R, Vasanji A, Anand-Apte B (2011). Increased neovascularization in mice lacking tissue inhibitor of metalloproteinases-3.. Invest Ophthalmol Vis Sci.

[35] Neale BM, Fagerness J, Reynolds R, Sobrin L, Parker M, Raychaudhuri S, Tan PL, Oh EC, Merriam JE, Souied E, Bernstein PS, Li BX, Frederick JM, Zhang K, Brantley MA, Lee AY, Zack DJ, Campochiaro B, Campochiaro P, Ripke S, Smith RT, Barile GR, Katsanis N, Allikmets R, Daly MJ, Seddon JM (2010). Genome-wide association study of advanced age-related macular degeneration identifies a role of the hepatic lipase gene (LIPC).. Proc Natl Acad Sci USA.

[36] Jonas JB, Tao Y, Neumaier M, Findeisen P (2012). Cytokine concentration in aqueous humour of eyes with exudative age-related macular degeneration.. Acta Ophthalmol (Copenh).

[37] Funk M, Karl D, Georgopoulos M, Benesch T, Sacu S, Polak K, Zlabinger GJ, Schmidt-Erfurth U (2009). Neovascular age-related macular degeneration: intraocular cytokines and growth factors and the influence of therapy with ranibizumab.. Ophthalmology.

[38] Haas P, Steindl K, Aggermann T, Schmid-Kubista K, Krugluger W, Hageman GS, Binder S (2011). Serum VEGF and CFH in exudative age-related macular degeneration.. Curr Eye Res.

[39] Gomi F, Ohji M, Sayanagi K, Sawa M, Sakaguchi H, Oshima Y, Ikuno Y, Tano Y (2008). One-year outcomes of photodynamic therapy in age-related macular degeneration and polypoidal choroidal vasculopathy in Japanese patients.. Ophthalmology.

[40] Chen H, Liu K, Chen LJ, Hou P, Chen W, Pang CP (2012). Genetic associations in polypoidal choroidal vasculopathy: a systematic review and meta-analysis.. Mol Vis.

[41] Lima LH, Schubert C, Ferrara DC, Merriam JE, Imamura Y, Freund KB, Spaide RF, Yannuzzi LA, Allikmets R (2010). Three major loci involved in age-related macular degeneration are also associated with polypoidal choroidal vasculopathy.. Ophthalmology.

[42] Zhang X, Wen F, Zuo C, Li M, Chen H, Wu K (2011). Association of genetic variation on chromosome 9p21 with polypoidal choroidal vasculopathy and neovascular age-related macular degeneration.. Invest Ophthalmol Vis Sci.

[43] Helgadottir A, Thorleifsson G, Magnusson KP, Gretarsdottir S, Steinthorsdottir V, Manolescu A, Jones GT, Rinkel GJ, Blankensteijn JD, Ronkainen A, Jaaskelainen JE, Kyo Y, Lenk GM, Sakalihasan N, Kostulas K, Gottsater A, Flex A, Stefansson H, Hansen T, Andersen G, Weinsheimer S, Borch-Johnsen K, Jorgensen T, Shah SH, Quyyumi AA, Granger CB, Reilly MP, Austin H, Levey AI, Vaccarino V, Palsdottir E, Walters GB, Jonsdottir T, Snorradottir S, Magnusdottir D, Gudmundsson G, Ferrell RE, Sveinbjornsdottir S, Hernesniemi J, Niemela M, Limet R, Andersen K, Sigurdsson G, Benediktsson R, Verhoeven EL, Teijink JA, Grobbee DE, Rader DJ, Collier DA, Pedersen O, Pola R, Hillert J, Lindblad B, Valdimarsson EM, Magnadottir HB, Wijmenga C, Tromp G, Baas AF, Ruigrok YM, van Rij AM, Kuivaniemi H, Powell JT, Matthiasson SE, Gulcher JR, Thorgeirsson G, Kong A, Thorsteinsdottir U, Stefansson K (2008). The same sequence variant on 9p21 associates with myocardial infarction, abdominal aortic aneurysm and intracranial aneurysm.. Nat Genet.

[44] Zuo C, Wen F, Li M, Zhang X, Chen H, Wu K, Zeng R (2012). COL1A2 polymorphic markers confer an increased risk of neovascular age-related macular degeneration in a Han Chinese population.. Mol Vis.

